# Focal brain lactate accumulation in metformin-induced encephalopathy without systemic lactic acidosis: A case report suggesting mitochondrial vulnerability in lentiform fork sign

**DOI:** 10.1016/j.ensci.2021.100383

**Published:** 2021-11-29

**Authors:** Akira Hanazono, Yoshiko Takahashi, Yui Sanpei, Sachiko Kamada, Masashiro Sugawara

**Affiliations:** Akita University Graduate School of Medicine, Department of Neurology, 1-1-1 Hondo, Akita 010-8543, Japan

**Keywords:** Metformin-induced encephalopathy, Lentiform fork sign, Chronic kidney disease, Lactate, Diabetes, Mitochondrial respiratory complex I

## Abstract

Metformin causes metabolic encephalopathy in some patients with end-stage chronic kidney disease, resulting in impaired consciousness and parkinsonism. This encephalopathy has a very characteristic magnetic resonance imaging feature in lentiform nuclei known as the “lentiform fork sign”. However, the mechanism is unknown. Here, we report a case of metformin-induced encephalopathy with a novel observation of lactate accumulation in the lentiform nuclei on magnetic resonance spectroscopy without systemic lactic acidosis. Since metformin is an inhibitor of mitochondrial complex-I, this focal brain lactate accumulation implies that a part of the pathogenesis of metformin-induced encephalopathy is the focal vulnerability of mitochondria to metformin in the lentiform nuclei. When metformin causes encephalopathy, not only testing for serum lactic acidosis and performing routine magnetic resonance imaging but also evaluation of brain lactate accumulation by magnetic resonance spectroscopy should be required to elucidate the etiology.

## Highlights

• Metformin-induced encephalopathy with lactate accumulation in lentiform fork sign was reported.
• Lentiform nuclei might be more vulnerable to metformin than other organs.
• This case suggested an etiology for the lentiform fork sign because metformin is a mitochondrial inhibitor.

## Graphical abstract

**Figure:**
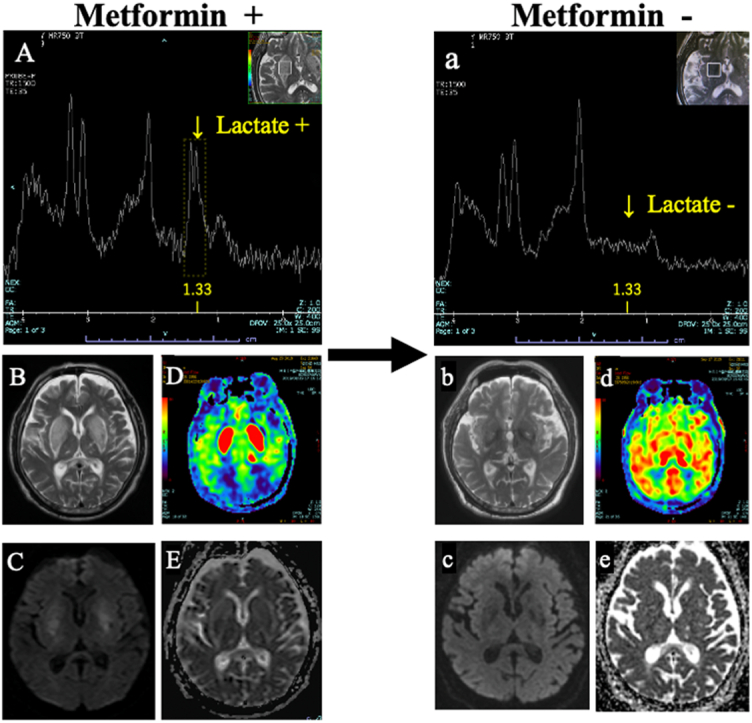

## 1 Introduction

Metformin causes metabolic encephalopathy in some patients with end-stage chronic kidney disease (CKD), resulting in impaired consciousness disturbance and parkinsonism [[1], [2], [3], [4], [5], [6]]. It is known as metformin-induced encephalopathy and has very characteristic magnetic resonance imaging (MRI) findings, known as the “lentiform fork sign”. However, the mechanism remains unclear because there are few case reports and no histopathological reports. Here, we report a case of metformin-induced encephalopathy with a novel finding of lactate accumulation in the lentiform nuclei on magnetic resonance spectroscopy (MRS) without systemic lactic acidosis, suggesting a part of the disease pathogenesis.

## 2 Case report

**Fig. 1:**
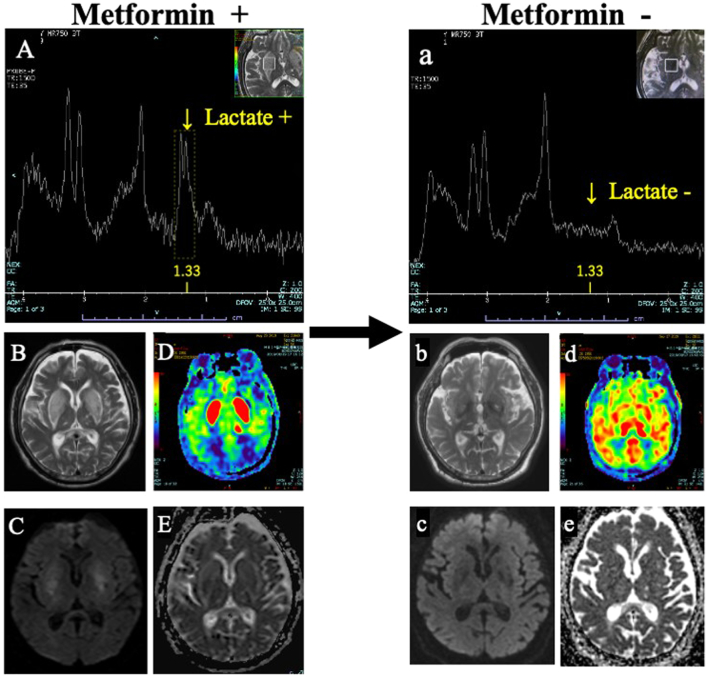
Lactate peaks with doublet are shown at 1.33 ppm on MRS with short echo time under metformin administration. Lactate peaks are not normally detected within the brain (A). Lentiform fork sign is shown in T2-weighted image (B). Focal hyperperfusion in bilateral lentiform nuclei is shown in ASL (D). DWI and ADC map show vasogenic and cytotoxic edema (C, E). All of these observations with metformin resolved after withdrawal (a, b, c, d, e). **Abbreviations**; MRS, magnetic resonance spectroscopy; ASL, arterial spin labeling; DWI, diffusion-weighted imaging; ADC, apparent diffusion coefficient.

A 63-year old male with an 8-year history of type 2 diabetes started hemodialysis 7 months before hospitalization. Diabetes mellitus was treated with teneligliptin 20 mg/day, acarbose 100 mg/day, pioglitazone 15 mg/day, and metformin 500 mg/day. The patient's most recent glycated albumin (GA) level was 16.7%. In previous institutions, these drugs, including metformin, were continued despite contraindication for hemodialysis patients. Later, anorexia with nausea gradually progressed, and he was admitted to our hospital because of acute drowsiness, akinesia, muscle rigidity, and a reduced gait stride (acute parkinsonism). At our institution, fever due to mild aspiration pneumonia was noted, but other vital signs, including blood pressure, were normal. A blood test revealed no abnormalities that could explain neurological symptoms, such as sodium, calcium, inorganic phosphorus, parathyroid hormone, blood sugar, ammonia, thiamin, cobalamin, ceruloplasmin, serum copper, antinuclear antibody, antitumor marker, and D-dimer. Cerebrospinal fluid (CSF) was also normal. Due to continuous conscious disturbance, MRI was performed on the day of admission, and it showed a typical “lentiform fork sign” on T2-weighted imaging. This bilateral edema of lentiform nuclei showed hyperperfusion of basal ganglia with arterial spin labeling (ASL) and mixed vasogenic and cytotoxic edema of lentiform nuclei on diffusion-weighted imaging (DWI) and apparent diffusion coefficient (ADC) maps (Fig. 1 B, C, D, E). In addition, MRS showed lactate accumulation (1.33 ppm doublet peak with short echo time) in the lentiform nuclei (Fig. 1 A) despite normal serum lactate levels (8.5 mg/dl) and absence of acidemia (pH 7.411 on blood gas analysis). These radiological findings were very similar to previous reports of metformin-induced encephalopathy with lentiform fork sign [[1], [2], [3],^5^,^6^]. Then, metformin was discontinued because there were no other drugs, such as methanol, ethylene glycol, or methamphetamine, that had previously been reported to produce a lentiform fork sign. Consciousness disorder, parkinsonism, and anorexia subsequently resolved within 3 days. A significant improvement was also shown on MRI at 3 weeks from disease onset (Fig. 1 a, b, c, d, e). Notably, the disappearance of the lactate peak after withdrawal (Fig. 1 a) suggested that focal anaerobic metabolic acidosis in lentiform nuclei due to metformin might have been the underlying etiology of the lentiform fork sign. With no other medical intervention other than drug discontinuation, spontaneous remission due to regular hemodialysis was very unlikely.

## 3 Discussion

There is a syndrome presenting peculiar edema of the lentiform nuclei in the brain known as “the lentiform fork sign”, which causes neurological disturbance such as consciousness disturbance and movement disorders like parkinsonism. Although the definition of lentiform fork sign was based on a radiological observation [7] and emerges in various diseases, this syndrome likely has some shared mechanism, because the radiological findings in the lentiform nuclei are so unique with the bilateral symmetrical basal ganglia T2 hyperintensities surrounded by a more hyperintense rim delineating the lentiform nuclei [[2], [3], [4], [5], [6], [7], [8], [9], [10], [11], [12], [13], [14]]. Because the most frequent shared backgrounds in lentiform fork signs were uremia and diabetes, “diabetic uremic syndrome” is the most frequent diagnostic name for lentiform fork signs. In addition, Asian patients and metabolic acidosis are also frequently reported related to this sign [9,11,13].

On the other hand, metformin-induced encephalopathy is one of the causes of the lentiform fork sign, and is characterized by “onset with metformin and improvement with withdrawal” [[1], [2], [3], [4], [5], [6]]. Antecedent appetite loss with nausea observed in our case might be a predictor of metformin's intoxication because the most frequent adverse event of the drug is gastrointestinal issues [2,5]. In addition, in a case report, two doses of metformin administered at different timies induced similar encephalopathies [4], suggesting a clear causal relationship with metformin. However, most patients on metformin do not develop encephalopathy even with CKD involvement (which implies individual differences in disease thresholds, and that metformin might be only one of multiple factors). In addition, this rare encephalopathy is not well known, and previous reports of metformin have underestimated the causal relationship. For example, metformin was used in four reported cases of lentiform fork sign in CKD patients, but there were no discussions about metformin despite the contraindication [[10], [11], [12],^15^]. Added to these underestimations, because recent reports recommended expanded metformin use in a subset of CKD patients [16,17], unreported metformin-induced encephalopathy might also expand to CKD patients.

In addition to these previous reports, our case was the first to show a causal relationship between metformin and lactate accumulation in the brain with a clear correlation with metformin withdrawal. Although the present case had no data on lactate concentration in CSF, a lactate peak on MRS is regarded as more sensitive evidence of lactate accumulation in the brain than CSF lactate [18]. In addition, because of the normal range of lactate in the blood, our case also suggested the higher vulnerability of lentiform nuclei to metformin than other organs. Therefore, our observation casts doubt on the aforementioned reports recommending expanded use of metformin in CKD patients [16,17], because they evaluated only “serum” lactate levels, but not those in the brain. On the other hand, we assumed metformin might be only one of multiple factors of lactic accumulation in the lentiform fork sign, because the lactate in the lentiform nuclei was also reported in CKD patients even without metformin use [11,13,14]. As mentioned above, the present case suggested that focal anaerobic metabolism (lactate accumulation) seems to be a fundamental shared mechanism of the lentiform fork sign, even if this MRI finding is shown in various diseases.

Pharmacologically, metformin is an inhibitor of mitochondrial respiratory complex I [19]. Interestingly, a hereditary mitochondrial disease with respiratory complex I deficiency showed similar lesions of the basal ganglia with a lactate peak in 90% of patients [20]. In addition, 1-methyl-4-phenyl-1,2,3,6-tetrahydropyridine (MPTP), a strong inhibitor of mitochondrial complex I and a common drug for making experimental animal models for Parkinson's disease, showed a quite similar brain edema of lentiform nuclei with lactate accumulation in cats [21]. Besides, uremia, which plays a major role in the lentiform fork sign, may cause mitochondrial respiratory complex dysfunction [22]. Furthermore, both hypo and hyperglycemia were reported to disturb mitochondrial function in the brain [23]. Based on these reports and our observation in the present case, we hypothesized that the accumulation of mitochondrial dysfunction, especially in respiratory complex I, might be an important shared key to the lentiform fork sign. We also assumed that metformin sometimes plays a role in exceeding the onset threshold, and thus is called “metformin-induced encephalopathy”, but mostly it acts as one of the provoking factors in the background.

In terms of limitations, the present report did not discuss hyperperfusion in the lentiform nuclei on ASL (Fig. 1. D), which was an interesting observation because a similar observation was reported in uremic patients with metformin [10], and without metformin [8,13]. Therefore, this focal vessel dilatation might be another shared observation of the lentiform fork sign and should be discussed more in other research.

## 4 Conclusion

This was the first report in which a causal relationship was observed between metformin and lactate accumulation in the brain, based on a clear correlation with metformin withdrawal. Based on pharmacology, this effect of metformin might be related to local mitochondrial dysfunction in the lentiform nuclei. When metformin is prescribed, the absence of systemic lactic acidosis alone is not sufficient to prove safety, because our findings suggest that lentiform nuclei might be more vulnerable to metformin. Therefore, if disturbed consciousness or parkinsonism develops during metformin use, not only routine MRI and “serum” lactic acidosis testing but also assessment of “brain” lactate accumulation in the lentiform nuclei by MRS should be required to prove the drug's safety and elucidate the etiology.

## Declaration of Competing Interest

None.
